# Identification and functional characterization of the *AGO1* ortholog in maize

**DOI:** 10.1111/jipb.12467

**Published:** 2016-04-07

**Authors:** Dongdong Xu, Hailong Yang, Cheng Zou, Wen‐Xue Li, Yunbi Xu, Chuanxiao Xie

**Affiliations:** ^1^ Institute of Crop ScienceChinese Academy of Agricultural SciencesBeijing100081China

**Keywords:** AGO1, functional complementation, maize, *ZmAGO1a*

## Abstract

Eukaryotic Argonaute proteins play primary roles in miRNA and siRNA pathways that are essential for numerous developmental and biological processes. However, the functional roles of the four *ZmAGO1* genes have not yet been characterized in maize (*Zea mays* L.). In the present study, *ZmAGO1a* was identified from four putative *ZmAGO1* genes for further characterization. Complementation of the *Arabidopsis ago1‐27* mutant with *ZmAGO1a* indicated that constitutive overexpression of *ZmAGO1a* could restore the smaller rosette, serrated leaves, later flowering and maturation, lower seed set, and darker green leaves at late stages of the mutant to the wild‐type phenotype. The expression profiles of *ZmAGO1a* under five different abiotic stresses indicated that *ZmAGO1a* shares expression patterns similar to those of Argonaute genes in rice, *Arabidopsis*, and wheat. Further, variation in *ZmAGO1a* alleles among diverse maize germplasm that resulted in several amino acid changes revealed genetic diversity at this locus. The present data suggest that *ZmAGO1a* might be an important *AGO1* ortholog in maize. The results presented provide further insight into the function of *ZmAGO1a*.

**Edited by:** Hailing Jin, University of California, Riverside, USA

**Abbreviations**
*35S*: *CaMV35S* promoter
AGO: Argonaute protein
Col 0: *Arabidopsis thaliana* (L.) ecotype Colombia
DUF: domain of unknown function
HMM: Hidden Markov Model
Low N: low nitrate
Low P: low phosphate
Mid: middle domain
miRNA: microRNA
MS: Murashige and Skoog nutrient
*Mu*: *Mu* transposon
NCBI: the National Center for Biotechnology Information
NJ: neighbor‐joining
PAZ: Piwi‐Argonaute‐Zwille
PEG: polyethylene glycol
piRNAs: Piwi‐interacting RNA
PPFD: photosynthetic photon flux density
qRT‐PCR: quantitative real time PCR
QTL: quantitative trait loci
RISC: RNA‐induced silencing complex
RNAi: RNA interference
siRNA: small interference RNAs
RNAs: short or small RNAs
wt: wild‐type

## INTRODUCTION

Argonaute (AGO) proteins are key mediators of all small‐RNA‐guided gene‐silencing processes involving ≈ 21–26 nt short RNAs (sRNAs) that are cleaved from double‐stranded or partially double‐stranded RNAs by the RNase III enzyme Dicer (Baulcombe 2004; Meister 2013). As key components of the RNA‐induced silencing complex (RISC), Argonaute proteins guide sRNAs to specific targets through sequence complementarity, leading to cleavage of target mRNA sequences, translational repression, or chromatin modification (Baumberger and Baulcombe 2005). Through their crucial roles in the regulation of eukaryotic gene expression, AGO proteins broadly participate in numerous biological processes, including developmental timing, cell differentiation, cell proliferation, cell death, metabolic control, immunity, transposon silencing, alternative splicing, and DNA repair, among other processes (Wei et al. 2012).

Argonautes are large proteins, approximately 90–100 kDa, consisting of one variable N‐terminal domain and conserved C‐terminal PAZ (Piwi‐Argonaute‐Zwille), Mid, and PIWI domains (Tolia and Joshua‐Tor 2007; Hutvagner and Simard 2008). AGO protein families generally can be classified into three groups in view of their phylogenetic relationships and capacity to bind to sRNAs (Vaucheret 2008). These include the AGO proteins that bind to miRNAs and siRNAs, the PIWI proteins that bind to piRNAs, and the proteins that bind to secondary siRNAs that have only been described in *C. elegans* (Yigit et al. 2006). As for crop plants, a total of 19 *Argonaute* genes in rice (*Oryza sativa*) and 15 in tomato (*Solanum lycopersicum*) have been identified (Kapoor et al. 2008; Bai et al. 2012). Among Argonaute proteins characterized so far, AGO1 plays essential roles in miRNA and siRNA pathways (Vaucheret 2008). AtAGO1 mediates cleavage of target mRNAs, which is the main proposed mechanism of plant miRNA action. The AtAGO1‐RISC complex has also been found to repress translation initiation (Iwakawa and Tomari 2013). Indeed, complementation analysis of *ago1* mutant plants revealed that the catalytic residues of AtAGO1 are required to restore the normal wild‐type phenotype to the *ago1* mutant, suggesting that the Slicer activity of AtAGO1 is essential for plant development (Carbonell et al. 2012). Complementation of *Arabidopsis ago1* mutants with genes from other higher plant species that also encode AGO1 and with the other alleles of *ZmAGO1* should be an effective way to examine the function of the *ZmAGO1* genes we identified.

The organization of genes encoding AGO1 in the genome differs among species, even though AGO1 plays important roles in similar important developmental, immunity, and other biological processes. For example, the *Arabidopsis* genome contains a single *AGO1* gene, but the protein it encodes shares some functional redundancies with other AGO proteins. For example, AtAGO10 shows functional redundancies with AGO1 during at least some aspects of development (Lynn et al. 1999). In contrast, four *AGO1* genes have been found in rice that exhibit some functional redundancies and specialization (Wu et al. 2009). In previous work on maize AGO proteins, 18 *AGO* genes were identified in the maize genome by Hidden Markov Model (HMM) analysis (Qian et al. 2011). The temporal‐spatial expression patterns of 17 of these *AGO* genes were analyzed to identify meiosis‐specific AGO proteins in maize. A meiosis‐expressed AGO protein (ZmAGO18b) was specifically expressed during meiosis in pollen mother cells in maize and was enriched in the tapetum and germ cells of meiotic anthers (Zhai et al. 2014). Maize AGO104 was found to be required for the generation of male and female spores through meiosis, and to act in repression of somatic fate in germ cells (Singh et al. 2011). Four maize *AGO1* genes have been predicted in other studies (Qian et al. 2011; Zhai et al. 2014), but it has been unclear whether these genes could be annotated as encoding functional AGO1 proteins. Here, we focused on *ZmAGO1a* to analyze its expression pattern in response to abiotic stress, its functional complementation of an *Arabidopsis ago1* mutant, and the DNA sequence variation in this gene among diverse maize germplasm. The data and results presented here provide further insights into the expression and function of the AGO1 protein and the sequence diversity within this gene in maize.

## RESULTS

### Identification of *Argonaute 1* genes in maize

**Figure 1:**
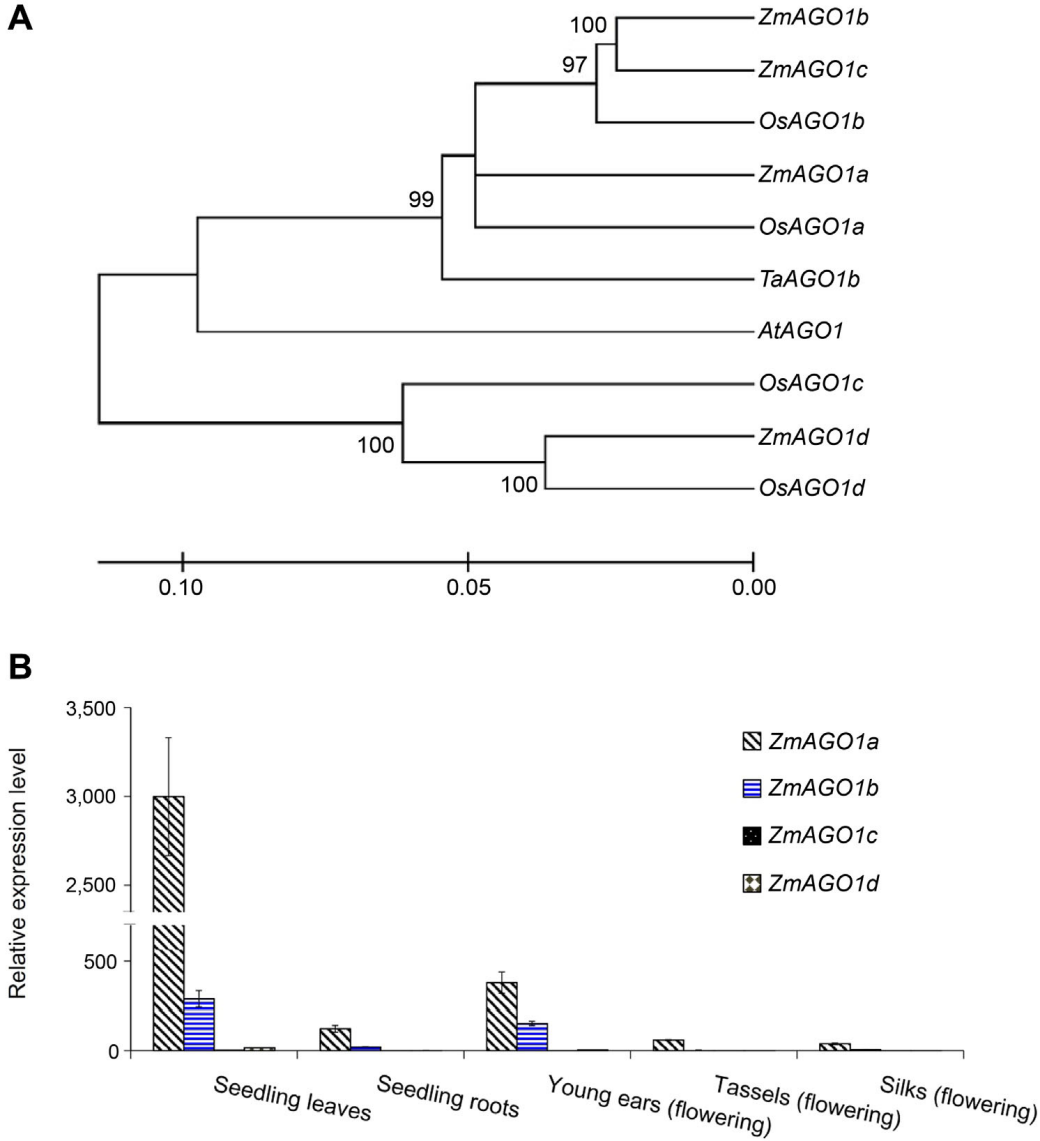
**Phylogenetic analysis (A) of four predicted *Zea mays AGO1* genes (*ZmAGO1a*‐*1d*) and their expression pattern (B) in selected organs in different developmental stages** **(A)** *AtAGO1*, *Arabidopsis* *AtAGO1*; *ZmAGO1a*,*1b*, *1c* and *1d* were *AGO1* genes in maize; *OsAGO1a, 1b,1c, 1d* were *AGO1* genes in rice; *TaAGO1b*, wheat *AGO1b*. **(B)** Seedling leaves, 3‐week maize seeding leaves; Seedling roots, 3‐week maize seeding roots; Young ear, maize immature ear in flowering; Tassels, maize flowering male inflorescence; Silks, maize flowering (silking) silks in young ear. Note: The *AGO1* gene accessions are listed in Table 1.

Four putative *Zea mays ZmAGO1* genes (Table 1), encoding both PAZ and PIWI domains, were predicted using an online DELTA‐BLAST tool against the maize genome (*Zea mays*, (TaxId:4577)) at http://blast.ncbi.nlm.nih.gov/Blast.cgi. The predicted results were consistent with previous studies (Qian et al. 2011; Zhai et al. 2014) that identified *GRMZM2G361518*, which had been designated *ZmAGO1d* by Qian et al. (2011), but was named *ZmAGO1f* by Zhai et al. (2014). The genomic location, intron/exon structure, and cDNA length of this gene have been reported previously (Qian et al. 2011; Zhai et al. 2014), but its genomic location has changed due to updates to the maize genome sequence. Based on the existing functional annotations, four *OsAGO1* genes from rice, one *AtAGO1* gene from *Arabidopsis*, and one *TaAGO1* gene from wheat were chosen for analysis of their phylogenetic relationships with the four predicted putative *ZmAGO1* genes. Phylogenetic analyses revealed a close evolutionary relationship among the predicted *Zea mays AGO1* genes with those from rice, wheat, and *Arabidopsis* (Figure 1A). Among these AGO‐encoding genes, those from monocot species, *ZmAGO1a*, *OsAGO1a*, and *TaAGO1b*, were clustered together, indicating their relatively closer evolutionary relationship. Motif Scan (See Materials and Methods.) was used to search for conserved motifs across these known and putative *AGO1* genes. Motif scanning revealed that these genes, except for *OsAGO1c* and *TaAGO1b*, all contain PIWI, PAZ, DUF1785, and Gly‐rich domains (Table 1). The four *AGO1* genes in rice appear to be functionally redundant in the miRNA pathway (Wu et al. 2009). The genomic organization, phylogeny, and conserved domains of the *ZmAGO1* genes suggested that the maize genes might share a function similar to that of the rice *AGO1* genes. The expression profiles of four *ZmAGO1* genes (Figure 1B) among different organs or tissues including seedling leaves and roots, young ear, pollinating tassels, and flowering silks revealed that *ZmAGO1a* had the highest expression levels across those observed organs or tissues, indicating it is the primary member of the families. In addition, the phylogenetic and expression profiling analyses suggest that functional characterization should focus initially on *ZmAGO1a*.

**Table 1:** Conserved motifs among *AGO1* gene homologs across maize, rice, wheat, and *Arabidopsis*

Gene name	Accession number	Amino acid sequence regions of conserved motifs					
		PIWI	PAZ	DUF1785	Gly‐rich	Gln‐rich	Pro‐rich
*AtAGO1*	AT1G48410	678–999	389–526	336–388	13–108	33–168	–
*ZmAGO1a*	GRMZM2G441583	728–1050	438–575	385–437	8–148	–	48–181
*ZmAGO1b*	AC209206.3_FG011	723–1042	424–570	371–423	8–132	180–201	–
*ZmAGO1c*	GRMZM2G039455	708–1029	418–555	365–417	13–126	–	–
*ZmAGO1d*	GRMZM2G361518	653–974	360–498	307–359	20–86	–	–
*OsAGO1a*	LOC_Os02g45070	709–1030	419–556	366–418	29–130	–	–
*OsAGO1b*	LOC_Os04g47870	746–1067	456–593	403–455	25–164	212–233	–
*OsAGO1c*	LOC_Os02g58490	638–959	351–485	298–350	–	32–66	–
*OsAGO1d*	LOC_Os06g51310	669–990	379–516	326–378	10–55	–	–
*TaAGO1b*	AGB34310.1	497–818	207–344	154–206	–	–	–

### Complementation of *Arabidopsis ago1‐27* with *ZmAGO1a*

**Figure 2:**
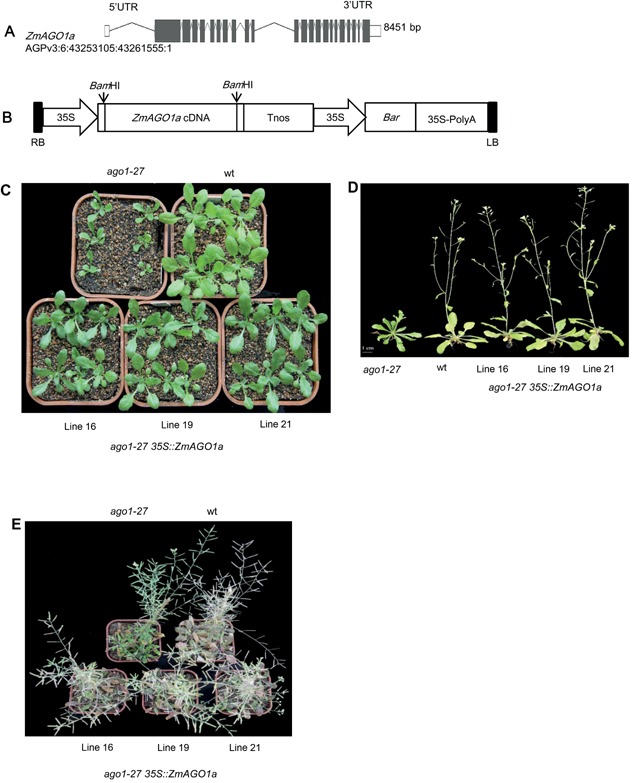
**The effect of constitutive expression of *ZmAGO1a* in *ago1‐27* of *Arabidopsis*** **(A)** *ZmAGO1a* gene structure. Empty rectangles, untranslated region (5′ or 3′ UTR); Black rectangles, exons; Fold lines, introns; 8451bp, total genomic DNA length; AGPv3:6:43253105:43261555:1, maize genome ver3 physical region annotation. **(B)** The *p35S::ZmAGO1a* construct in which the *ZmAGO1a* cDNA was expressed under the control of *CaMV35S* promoter (*35S*) and *NOS* terminator (*Tnos*). Hollow arrows with the text “35S” inside indicated *CaMV35S* promoter. *Bar*, glufosinate ammonium resistance selection marker; *35S‐PloyA*, *CaMV35S polyA* signal sequence; LB and RB, T‐DNA left and right borders. Vertical arrows indicated the restriction sites. **(C)** Phenotypic comparison of seedlings among the wild type, *ago1‐27* and *ago1‐27* carrying *p35S::ZmAGO1a* construct. **(D)** Phenotypic comparison at the flowering stage among the wild type, *ago1‐27* and *ago1‐27* carrying *p35S::ZmAGO1a* construct (note: the ago mutant flowered later). **(E)** Phenotypic comparison at the mature state among the wild type, *ago1‐27* and *ago1‐27* carrying *p35S::ZmAGO1a* construct (note: the ago mutant matured later.) **C**–**E**: wt, wild type *Arabidopsis thaliana* (L.) Colombia (Col‐0); *ago1‐27*, *Arabidopsis thaliana* (L.) *ago1‐27* mutant in the Col‐0 background; line 16, 19, and 21: three independent homozygous *ago1‐27* T3 lines transformed with *35S::ZmAGO1a*.

*ZmAGO1a* has a gene structure typical of *AGO1* genes (Figure 2A). The full‐length *ZmAGO1a* cDNA from Ye478, an important temperate inbred maize line from China, was isolated to construct a constitutive overexpression vector driven by the *CaMV35S* (35S) promoter (Figure 2B) in an *Arabidopsis ago1‐27* mutant in the Columbia (Col) background (Figure 2C). Plants derived from three independent transformation events, lines 16, 19 and 21, the *Arabidopsisago1‐27* mutant, and *Arabidopsis* Col wild‐type (wt) plants were grown in the same culture medium and under the same conditions (Liu et al. 2014) to compare their phenotypes (Figure 2C, D, E). As in a previous study (Morel et al. 2002), the *ago1‐27* mutant developed smaller rosettes with darker green, serrated, narrower leaves; initiated flowering in 7–12 d; and produced around ≈ 50% of the seeds with typical wild type (Figure 2C, D, E; Table 2). At the seedling stage (Figure 2C), *ZmAGO1a* overexpression lines had larger leaves with shallower serrated leaf edges compared to the *ago1‐27* mutant, but its leaf color was darker green than that of wild type (Figure 2C; Table 2). All *35S::ZmAGO1a‐*transformed lines had significant differences in leaf length and width from the *ago1‐27* mutant, indicating larger leaves and rosettes than in the mutant (Table 2). As shown by the leaf length/width ratio, the mutant had narrower leaves than the wild type and the *35S::ZmAGO1a*‐transformed lines. Most of the phenotypic traits of the *35S::ZmAGO1a‐*transformed lines, except for leaf color at the seedling stage, were comparable to those of wild type. However, the darker green leaf color disappeared at the flowering stage (Figure 2D; Table 2). *ZmAGO1a* overexpression lines 16, 19, and 21 appeared more similar to wild type at flowering time in terms of plant height, leaf number, and leaf color. However, these lines matured about 12 d earlier, and had higher silique numbers per plant and fertile seeds compared with the *ago1‐27* mutant (Figure 2D, E; Table 2). Thus, constitutive overexpression of *ZmAGO1a* in the *ago1‐27* mutant could nearly rescue the hypomorphic*ago1‐27* mutant phenotype.

**Table 2:** Complementation by constitutive overexpression of *ZmAGO1a* in *Arabidopsis ago1‐27* mutant

Line	Genotype	Phenotypes on leaves						Growing days (d)		Silique and seed nos. per plant	
		Leaf No.	Margin serration	Stages with dark green leaves	Length (cm)	Width (cm)	Length/width	Days to flower	Days to maturity	Silique count	Seed count
*ago1‐27*	Columbia *AtAGO1* mutant	7	+++	vegetative and reproductive stages	1.14 ± 0.03 C*	0.53 ± 0.02 C*	2.2 ± 0.09 A*	wt + 12	wt + 12	32.4 ± 1.83 B*	1458 ± 82.5 B*
16	*ago1‐27* with *35S::ZmAGO1a*	10	+	only at vegetative stage	1.34 ± 0.03 B*	0.80 ± 0.02 B*	1.7 ± 0.05 B*	wt + 2	Wt + 1	46.4 ± 1.50 A*	2088 ± 67.6 A*
19	*ago1‐27* with *35S::ZmAGO1a*	10	+	only at vegetative stage	1.49 ± 0.03 A*	0.92 ± 0.03 A*	1.6 ± 0.03 B*	wt + 1	Wt + 1	46.6 ± 1.40 A*	2097 ± 63.0 A*
21	*ago1‐27* with *35S::ZmAGO1a*	10	+	only at vegetative stage	1.50 ± 0.04 A*	0.90 ± 0.02 A*	1.7 ± 0.02 B*	wt + 1	Wt + 1	47.4 ± 0.87 A*	2133 ± 39.2 A*
wt	Columbia C0 (wild type)	11	−	Control	1.58 ± 0.03 A*	1.00 ± 0.02 A*	1.6 ± 0.04 B*	wt	wt	48.0 ± 3.89 A*	2160 ± 174.9 A*

“*” Values followed by different letters are significantly different according to Tukey's HSD (Honestly Significant Difference) test at a significance level of 0.05.

**Figure 3:**
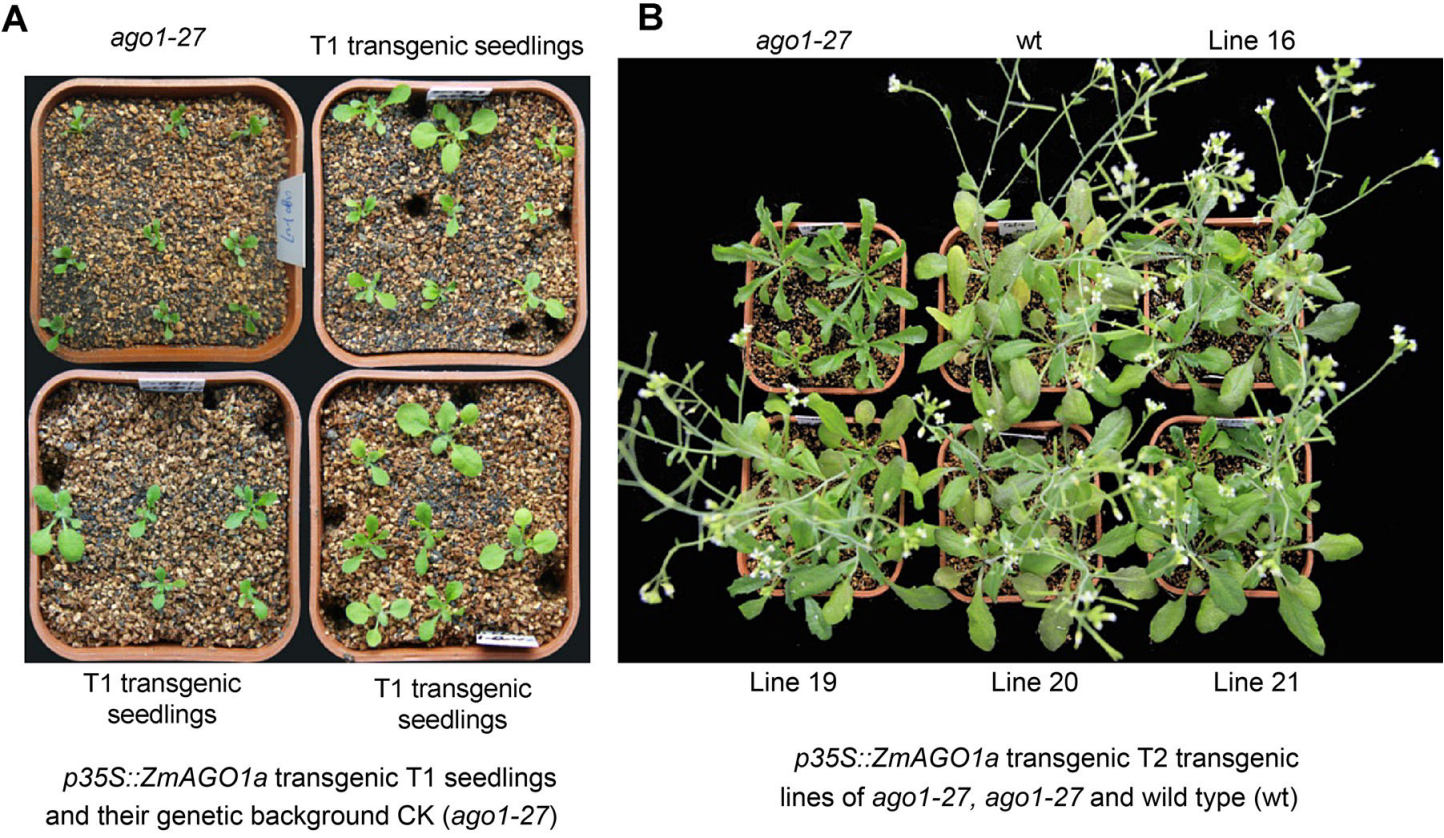
**Overexpression of *ZmAGO1a* in *Arabidopsisago1‐27* (T1 and T2 generations) and its rescue effect on phenotype** (**A)** *p35S::ZmAGO1a* transgenic T1 seedlings and their genetic background CK (*ago1‐27*). **(B)** Phenotyping comparison of T2 transgenic lines (line 16, 19, 20, and 21) at genetic background of *ago1‐27* with *ago1‐27* and wild type (wt. Col‐0). *ago1‐27* was also Col‐0 background but with *ago1* mutation.

To provide a full view of the functional complementation of *Arabidopsis* *ago1‐27* by *ZmAGO1a*, profiles of the restored phenotypes of transformants from the T1 and T2 generations are shown in Figure 3. Of ∼ 2,300 transformed T1 seeds screened, 21 bialaphos‐resistant plants were recovered and confirmed for the presence of the integrated transgene using polymerase chain reaction (PBR). The phenotypes of eight of 21 T1 lines were fully rescued (Figure 3A), and 13 of them exhibited partially restored phenotypes for leaf color, leaf morphology, or both (Figure 3A). The phenotype of four T2 lines (Figure 3B) including three selected T3 homozygous lines (Figure 2C, D, E) was well restored. The result shows that the functional complementation was quite stable both within individual lines and across generations.

### Expression of *ZmAGO1a* among *ZmAGO1a* transformants and expression of endogenous *AtAGO1* in the *ago1‐27* mutant and wild type

**Figure 4:**
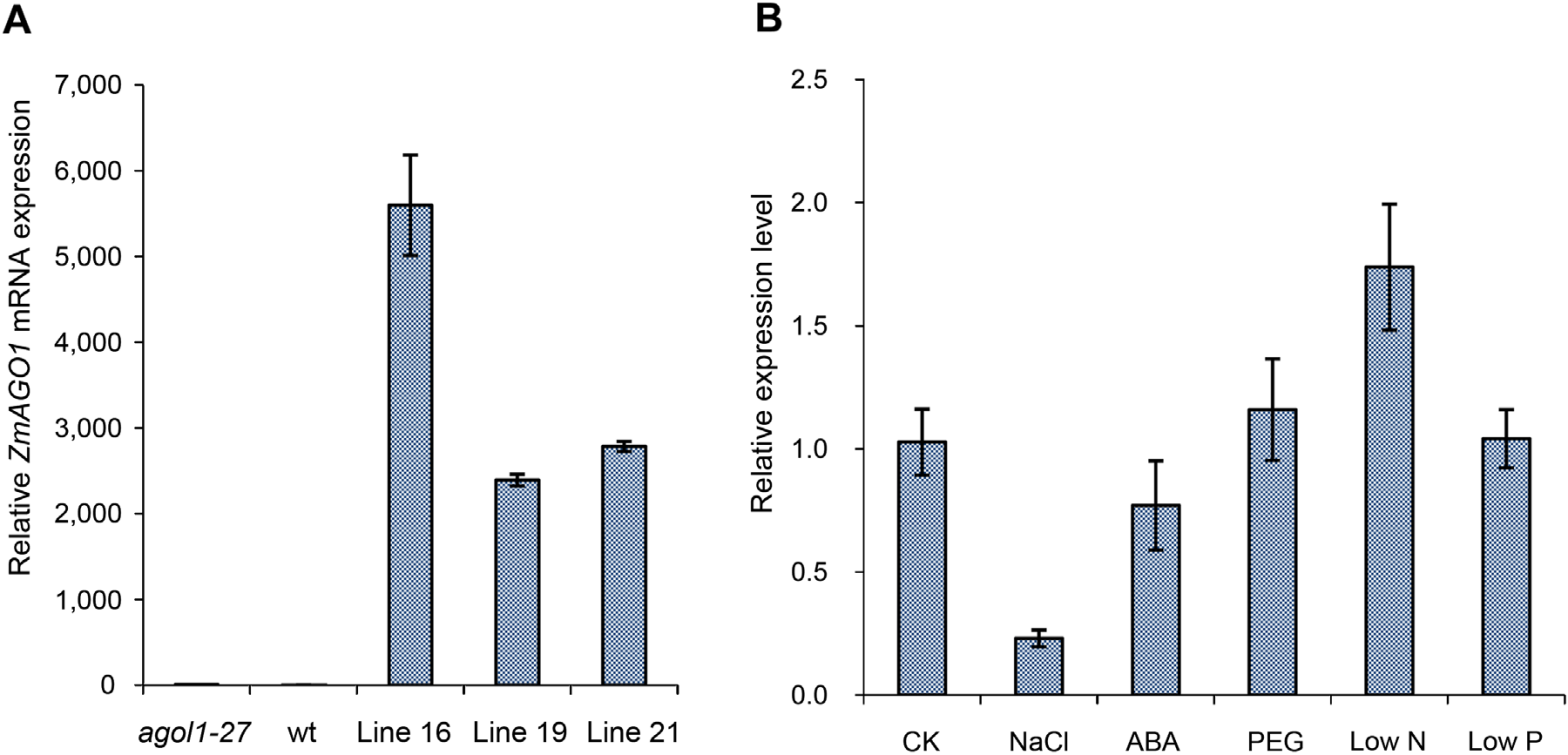
**Relative expression levels of *ZmAGO1a* (A) in different *Arabidopsis* genotypes and relative expression level of *ZmAGO1a* in response to different abiotic stresses in 3‐weeks‐old maize seedling leaves** **(A)** wt, wild type *Arabidopsis thaliana* (L.) Colombia (Col‐0); *ago1‐27*, *Arabidopsis thaliana* (L.) *ago1‐27* mutant in the Col‐0 background; lines 16, 19, and 21, three independent *ago1‐27* lines transformed with *35S::ZmAGO1a*. **(B)** CK, control; NaCl, salt stress (NaCl, 200 mM); ABA, 100 mΜ ABA; PEG, dehydration (PEG‐6000; 20% w/v); LN, low nitrate (0.04 mM NO_3_^−^); LP, low phosphorus (2.5 µM KH_2_PO_4_).

To analyze the expression of *ZmAGO1* and native *AtAGO1*, quantitative analyses of relative mRNA expression levels of the *AGO1* gene were performed on each independent *ZmAGO1a* transformant *Arabidopsis* line, and on the *Arabidopsis ago1‐27* mutant and wild type. The expression of *ZmAGO1* relative to *alpha‐Tubulin 4*, the internal reference gene, was over 2000‐fold higher in the *Arabidopsis ZmAGO1a* transformant lines 16, 19, and 21, compared to the same internal reference gene in the *ago1‐27* mutant and wild‐type *Arabidopsis* (Figure 4A). These results indicate that the *ZmAGO1a* transgene was expressed at high levels across different tissues when transformed into the *Arabidopsis ago1‐27* background. However, the expression of endogenous *AtAGO1* was 3.7‐fold higher in *ago1‐27* than in Col wild type (Figure S1). This result was consistent with a previous study suggesting that the single‐nucleotide mutation in *AtAGO1* results in compensatory overexpression of the *AtAGO1a* mRNA and AtAGO1 protein in the *ago1‐27* background (Mallory et al. 2009). Such mRNA overexpression was also observed among the *Arabidopsis ZmAGO1a* overexpression lines.

### Expression profiles of *ZmAGO1a* in response to diverse abiotic stresses in maize

To generate an expression profile of *ZmAGO1a* to analyze the role(s) of this gene during abiotic stress responses, five diverse abiotic stresses, including dehydration (polyethylene glycol (PEG) treatment), salt (NaCl), ABA, low nitrate (low N) and low phosphate (low P), were applied at the seedling stage as described in Materials and Methods. Young leaves, the organs with the highest level of *ZmAGO1a*expression at this stage, were chosen for analysis of *ZmAGO1a* expression in response to these abiotic stresses.

*ZmAGO1a* was differentially expressed in leaves under different stress conditions. *ZmAGO1a* expression was slightly upregulated under low N, low P, and PEG treatment, increasing by 1.74‐, 1.04‐, and 1.16‐fold, respectively, (Figure 4B). A previous analysis in rice indicated that expression of the *OsAGO1a* and *OsAGO1b* genes was also slightly upregulated in seedlings under various abiotic stresses (Kapoor et al. 2008). Our results indicate that *ZmAGO1a* expression was obviously downregulated under salt stress (NaCl), while the previous study indicated that expression of both *OsAGO1a* and *OsAGO1d* were slightly downregulated in rice under salt and drought stresses (Kapoor et al. 2008). However, the effects of NaCl and PEG stress treatments on the expression of *ZmAGO1a* revealed in this study were consistent with those of previous studies in maize (Qian et al. 2011).

### DNA sequence variation in *ZmAGO1a* genes

To gain insight into the diversity of the *ZmAGO1a* gene among lines within *Zea mays*, sequence variation in the *ZmAGO1a* gene among 87 lines representing both temperate and tropical maize germplasm was analyzed. In total, 105 SNPs were detected (Table S1). Most of the identified SNPs, 79 out of 105, were located in introns; however, 26 SNPs were located in exons (Table 3). Seven of 26 SNPs located in coding regions could result in amino acid changes. The allele frequencies of these variants, including those that resulted in protein sequence changes and those that did not, ranged from 5.75% to 36.78%, indicating that these variants were not rare alleles.

**Table 3:** DNA sequence variation in the coding region of *ZmAGO1a* among resequenced 87 lines representing broad tropical and temperate maize germplasm

Mutation type	DNA site	SNP	Amino acid site	Domain	Amino acid change	Nos. of variants (Tropical/Temperate)	Variant frequencies
Missense	1567	G‐A	8	Gly‐rich	G 8 D	5 (5/0)	5.75%
	1828	G‐A	95	Gly‐/Pro‐rich	R 95 H	32 (11/21)	36.78%
	1873	G‐T	110	Gly/Pro‐rich	G 110 V	7 (3/4)	8.05%
	2118	G‐C	192	–	A 192 P	8 (3/5)	9.20%
	6348	G‐T	770	PIWI	Q 770 H	32 (11/21)	36.78%
	6821	A‐G	870	PIWI	T 870 A	32 (11/21)	36.78%
	7219	G‐C	925	PIWI	E 925 Q	7 (3/4)	8.05%
	**Subtotal of missense mutation**					**123 (47/76)**	
Synonymous	1829	T‐C	95	Gly‐/Pro‐rich	–	21 (16/5)	24.14%
	2015	A‐G	157	Pro‐rich	–	18 (14/4)	20.69%
	2288	T‐C	248	–	–	21 (16/5)	24.14%
	2294	G‐T	250	–	–	29 (20/9)	33.33%
	2410	G‐A	263	–	–	32 (11/21)	36.78%
	2545	A‐G	308	–	–	21 (16/5)	24.14%
	2584	A‐G	321	–	–	29 (20/9)	33.33%
	2605	A‐C	328	–	–	29 (20/9)	33.33%
	3001	T‐G	408	DUF1785	–	29 (20/9)	33.33%
	6046	C‐T	695	–	–	29 (20/9)	33.33%
	6158	C‐T	733	PIWI	–	28 (19/9)	32.18%
	6270	C‐T	744	PIWI	–	32 (11/21)	36.78%
	6273	A‐G	745	PIWI	–	28 (20/8)	32.18%
	7245	T‐C	933	PIWI	–	32 (11/21)	36.78%
	7447	A‐G	970	PIWI	–	32 (11/21)	36.78%
	8018	A‐G	1071	–	–	29 (20/9)	33.33%
	8024	G‐A	1073	–	–	29 (20/9)	33.33%
	8048	G‐A	1081	–	–	21 (16/5)	24.14%
	8075	C‐T	1090	–	–	32 (11/21)	36.78%
	**Subtotal of synonymous mutation**					**521 (312/209)**	
					**Total**	**644 (359/285)**	

Note: the reference allele for both DNA and protein sequence numbering was B73 *ZmAGO1a*.

## DISCUSSION

### Possible functional redundancy of *ZmAGO1* genes

In contrast with *Arabidopsis*, which has a single *AGO1* gene, maize possesses four *AGO1* homologs, as does rice (Wu et al. 2009). Five maize *AGO1* genes were identified based on HMM analysis of sequences coding for conserved PAZ and PIWI domains in a previous report (Qian et al. 2011). However, our analysis and another parallel analysis (Zhai et al. 2014) with a more stringent model indicated that only four of the maize *AGO1* genes likely have biological activity. Consistent with previous reports (Qian et al. 2011; Zhai et al. 2014), our phylogenetic analysis showed that the four *ZmAGO1* homologs were highly conserved and were closely related to other rice and wheat *AGO1* homologs (Figure 1). Both some redundancy and some possible functional specialization among *AGO1* genes in rice has been suggested by sequencing miRNAs identified by gel retardation assays of AGO1 immunoprecipitates from the *in vitro* interaction of AGO and miRNAs (Wu et al. 2009). However, the specialized functions and biological roles of each particular AGO1 are still unknown in maize. Our expression profiles data showed that the *ZmAGO1a* was the highest expression in diverse organs, suggesting its' primary role among family members (Figure 1B). The phylogeny and similar organization of AGO1‐encoding homologs in rice and maize suggest that functional redundancy of *ZmAGO1* genes might be conserved. Among the four maize *AGO1* genes, *ZmAGO1a*, which is very closely related to *ZmAGO1b* and *ZmAGO1c* and more conserved with *AtAGO1* and *OsAGO1*, was selected for further analysis of gene expression and complementation analysis.

### *ZmAGO1a* rescues phenotypic defects and gene expression of *Arabidopsis ago1* mutants

In this study, we observed that constitutive overexpression of *ZmAGO1a* could rescue most of the phenotypic defects of the *ago1‐27* mutant, including rosette size, leaf size, leaf shape, flowering time, maturation time, and seed set (Figure 2). Some mutant phenotypes, such as leaves that were darker green and more serrated than wild type, could be partially restored to normal green color at later stages and shallower serrated leaf edges (Figure 2). The partially rescued phenotypes observed in these experiments could have been due to expression patterns specific to the promoter used. In our transformants, the *ZmAGO1a* gene was driven by the *CaMV35S* promoter so that it would be highly and constitutively expressed (Figure 4). In the same organ and tissue, the expression of endogenous *AtAGO1* was also 3.7‐fold higher in the *ago1‐27* mutant and 3.0‐fold higher in the *ZmAGO1a* overexpression transformants of *ago1‐27* than in wild‐type (Col‐0) *Arabidopsis*. Our results indicated that constitutive overexpression of *ZmAGO1a* could rescue most of the defects in the mutant phenotype, but could not rescue the impaired expression of native *AtAGO1*. So, AtAGO1 and ZmAGO1a in the *ZmAGO1a* overexpression transformants in *ago1‐27* might share some but not all functional roles. The unusual expression of these genes may or may not have any effect on their downstream functions, unless a direct effect on their own expression is suggested. The level of *AtAGO1* mRNA is maintained and modulated by feedback regulation of miR168 (Vaucheret et al. 2006). Obviously, a trans‐modulation element could affect the expression of the endogenous *AtAGO1* gene, but not that of exogenous *ZmAGO1a*, due to the absence of the effector within the expression cassette.

### Variation in *ZmAGO1a* sequences in maize

The functional basis of the genetic control of traits by quantitative trait loci (QTL) is due mostly to variation within numerous genes in a species like maize, which is one of the most diverse crops at both the molecular and phenotypic levels (Buckler et al. 2006). Such variation can occur within genes of interest in crop plant populations that have been subjected to both natural and artificial selection for a long time. Sequencing of 87 tropical and temperate maize lines at 10× depth was performed to analyze possible variation in the *ZmAGO1a* DNA sequence among these lines. Some missense mutations were found among these lines (Table 3), suggesting that *ZmAGO1a* could possibly contribute to phenotypic diversity in maize, as the function of AGO1 also contributes to the phenotype of other grass species such as rice (Wu et al. 2009).

### Reverse genetic analysis of *ZmAGO1a*

A reverse genetics approach, which seeks to draw conclusions from the phenotypes that arise after mutation of a gene of interest, as by knockout *via* gene tagging or knockdown through RNA interference (RNAi), could provide solid biological evidence for functional redundancy of *ZmAGO1a*. For example, rice *AGO1* genes have been knocked down using RNAi (Wu et al. 2009). By screening a public *Mu* transposon‐tagged maize library (Settles et al. 2007), we recovered one line (seed stock: UFMu‐04885) in which the 3′‐UTR region of *ZmAGO1a* was tagged. No obvious phenotype was evident in this line after the line was selfed until the *Mu*‐tagged *ZmAGO1a* allele was homozygous (data not shown). After obtaining this Mu‐tagged *ZmAGO1a* allele, more independent knockouts of each of the other *AGO1* genes will be required to further test the proposed functional redundancy of *ZmAGO1a* and the other *AGO1* genes in maize.

## CONCLUSIONS

The Argonaute1 (AGO1) protein plays key roles in miRNA and siRNA pathways that are essential for plant developmental and biological processes. We performed bioinformatics searches for maize genes encoding proteins containing conserved motifs consistent with AGO1 function and cloned the *ZmAGO1a* cDNA. The AGO1 function of *ZmAGO1a* was confirmed by complementation of the *Arabidopsis ago1‐27* mutant phenotype with a *ZmAGO1a* transgene that restores the mutant phenotype to wild type. The expression pattern of this gene and that of the endogenous *AtAGO1a* gene in response to various abiotic stresses indicated that *ZmAGO1a* might play an important role in both development and responses to environmental change as a member of the AGO gene family in maize.

## MATERIALS AND METHODS

### Plant materials, culture, and stress treatment conditions

*Arabidopsis thaliana* (L.) ecotype Colombia (Col‐0) was used as the wild type. The mutant *ago1‐27* in the Col ecotype, which developed rosettes with dark green and serrated leaves, had been described previously. The rosette size of this mutant is intermediate between that of stronger alleles and wild‐type plants, and this mutant initiates flowering 7–12 d after the wild type and is fertile (Morel et al. 2002).

After seed sterilization, plants were grown in sterile in 0.5× Murashige and Skoog (MS) nutrient agar medium in a short‐day (8 h light/16 h dark) regimen under a PPFD (photosynthetic photon flux density) of 70 µmol/ m^2^/s and constant temperature of 23 ± 1 °C. After 10 d, the plants were transferred to soil in a growth chamber with 16 h light: 8 h dark at 23 ± 1 °C.

Maize (*Zea mays* L. inbred lines B73 and Ye478) plants were grown in a greenhouse under 16 h light: 8 h dark at 28 °C (Xu et al. 2011). Three‐week‐old seedlings were subjected to one of the five following abiotic stress treatments: low phosphorus (2.5 µM KH_2_PO_4_), low nitrogen (0.04 mM NO_3_^–^), salt stress (NaCl, 200 mM), dehydration (polyethylene glycol, PEG‐6000; 20% w/v), or ABA (100 mΜ). Seedling leaves were sampled at 24 h after the treatment. The controls were treated with normal nutrient solution with no additives as listed (Xu et al. 2011).

### Bioinformatic analyses and identification of *Argonaute1* genes

AtAGO1 protein sequences from the MaizeGDB database (http://www.maizegdb.org/) and the National Center for Biotechnology Information (NCBI) were used to perform blastp queries against the maize (*Zea mays*, (TaxId:4577)) NR (Non‐redundant protein sequence)) database at NCBI using the newly developed algorithm DELTA‐BLAST (Domain Enhanced Lookup Time Accelerated BLAST). Amino acid alignments were conducted using DNAman software (LynnonBioSoft, Vaudreuil, Quebec, Canada). A phylogenetic tree was constructed based on this amino acid alignment result using the neighbor‐joining (NJ) method in MEGA5.05 (http://www.megasoftware.net/) with a Poisson model, pairwise deletion, uniform rates among sites, and 1000 bootstrap replicates. Protein domains were analyzed using Motif Scan (http://hits.isb-sib.ch/cgi-bin/motif_scan).

### Cloning the full‐length *ZmAGO1a* cDNA and construction of the overexpression vector

The primers for *ZmAGO1a* cDNA isolation were designed using Primer 5.0 (Premier Biosoft International, Palo Alto, CA, USA). The sense and antisense primers were 5′‐ggactctagaggatccCCCTTTCTCCGTCCGCAT‐3′ and 5′‐cggtacccggggatccCCCATCTTAATAGGAGTATATCGCA‐3′, respectively. PCR amplifications were performed using the HiFidelity PCR Kit (Tiangen Biotech Co., Ltd. Beijing, China). The temperature cycling program was 95 °C for 5 min, followed by 35 cycles of 95 °C for 15 s, 58 °C for 10 s, and 68 °C for 2 min, with a final extension at 68 °C for 10 min. PCR products were visualized on 1.0% agarose gels by ethidium bromide staining. The amplified fragment was subcloned into the CPB vector using the In‐Fusion HD Cloning Kit (Clontech Laboratories Inc., Mountain View, CA, USA). The vector was then isolated from *Escherichia coli* strain DH5α and transferred into *A. tumefaciens* strain GV3101 using the freeze‐thaw method (Clough and Bent 1998). The DNA sequence of the *ZmAGO1a* insertion in the plasmid construct in both *E. coli* and *A. tumefaciens* were verified by sequencing.

### Plant transformation and identification of transformants

*Arabidopsis* was transformed using the *Agrobacterium* floral dip method following a reported method (Clough and Bent 1998). Plants of the mutant *ago1‐27* in the Col‐0 background were used for transformation. Transgenic plants were screened on plates with 50 µg/mL glufosinate ammonium. The glufosinate‐resistant transformed plants were validated by PCR and with a strip test for detecting the bialaphos resistance protein encoded by the *bar* gene (QuickStix Kit for Liberty Link (*bar*) Cotton, EnviroLogix, Portland, ME, USA). T3 homozygous lines were used for all phenotyping and gene expression analysis.

### Gene expression analysis

Quantitative (q)RT‐PCR primers were designed using Beacon Designer7 software (Premier Biosoft International, Palo Alto, CA, USA), and qRT‐PCR was performed using the Applied Biosystems 7500 Fast Real‐Time PCR System with the SYBR *Premix Ex Taq* II Master Mix (Takara Bio Inc., Dalian, China). The primers for analyzing *ZmAGO1a* expression profiles were 5′‐GCTTGACTTGCTGATGGTAATACT‐3′ and 5′‐CTGATGCTTGTTCGCCTTGA‐3′. The primer pairs for *ZmAGO1b*, *ZmAGO1c* and *ZmAGO1d were* 5′‐GCCCGAGGTCACGAAATAC‐3′ and 5′‐GGGTCCTGCCATACTTTGA‐3′, 5′‐TTCTATTGATGGTGCCTGTGC‐3′ and 5′‐CAGACAGAAGGCAAGCAAAACG‐3′, 5′‐TCCAGAAACGCCACCATA‐3′ and 5′‐ACGACAGTTCCAGGAAGTAT‐3′, respectively. The primers used to amplify *GAPDH*, the internal reference for expression analysis in maize, were 5′‐ACTGTTCATGCCATCACTGC‐3′ and 5′‐GAGGACAGGAAGCACTTTGC‐3′. Amplification was followed by a melting curve analysis and an appropriate blank was applied. The primers used to amplify *AtAGO1* were 5′‐ATCAGACAGTGGCTCAAT‐3′ and 5′‐GACACGCTTCACATTCTC‐3′. The primers used to amplify *Arabidopsis alpha‐Tubulin 4*, the internal reference for expression analysis in *Arabidopsis*, were 5′‐GAGGGAGCCATTGACAACATCTT‐3′ and 5′‐GCGAACAGTTCACAGCTATGTTCA‐3′. The expression of both *ZmAGO1a* and *AtAGO1* was analyzed in the mutant, wild type, and transformants using qRT‐PCR. The 2^‐ΔΔCT^ method was used to estimate fold changes in the expression of each gene (Livak and Schmittgen 2001). Each data point represents four biological replicates with three technical replicates.

### Analysis of DNA sequence variation in *ZmAGO1a* among maize populations

The resequencing data (mean Poisson‐distributed 10 × depth for each line) from 87 maize inbreds representing tropical (47 of 87) and temperate (40) lines (Table  S1) were used to analyze DNA sequence variation in *ZmAGO1a* across lines. The data, SNP‐calling methods, and maize lines in our germplasm panel were described in our previous report (Xie et al. 2013). The resequencing data for *ZmAGO1a* have been deposited into GenBank at the NCBI under the accession numbers ranging from KJ958272 to KJ958358.

### PCR confirmation of *Mu* insertions

The *Mu* insertion mutations (UFMu‐04885) in *ZmAGO1a* were submitted to the MaizeGDB database (http://www.maizegdb.org/documentation/uniformmu/). Confirmation of *Mu* insertions was performed according to McCarty et al. (2005), including primer sequences.

## COMPETING INTERESTS

The authors declare no competing interests.

## AUTHOR CONTRIBUTIONS

D.X. carried out the experiments. C.X. designed the study and analyzed data. H.Y. carried out transformation. Z.C. performed DNA sequence variation analysis. W.X.L. and Y.X. performed data analysis. D.X. drafted the paper, and C.X. and Y.X. wrote and revised the paper. All authors read and approved the final manuscript.

## Supporting information

Additional supporting information may be found in the online version of this article at the publisher's web‐site.

**Table S1**. Maize lines resequenced for analysis of DNA sequence variation

**Figure S1**. Relative expression levels of native *AtAGO1* among wild type and *ZmAGO1a* transformed lines
