# Supplementary material for: Identification and functional characterization of the AGO1 ortholog in maize
Source: J Integr Plant Biol. 2016 Apr 7;58(8):749–58. doi: 10.1111/jipb.12467 (PMC5071735; doi:10.1111/jipb.12467)
Supplement: Supplementary file 2 — Figure S1. Relative expression levels of native AtAGO1 among wild type and ZmAGO1a transformed lines [file JIPB-58-749-s002.pptx]

## Slide 1
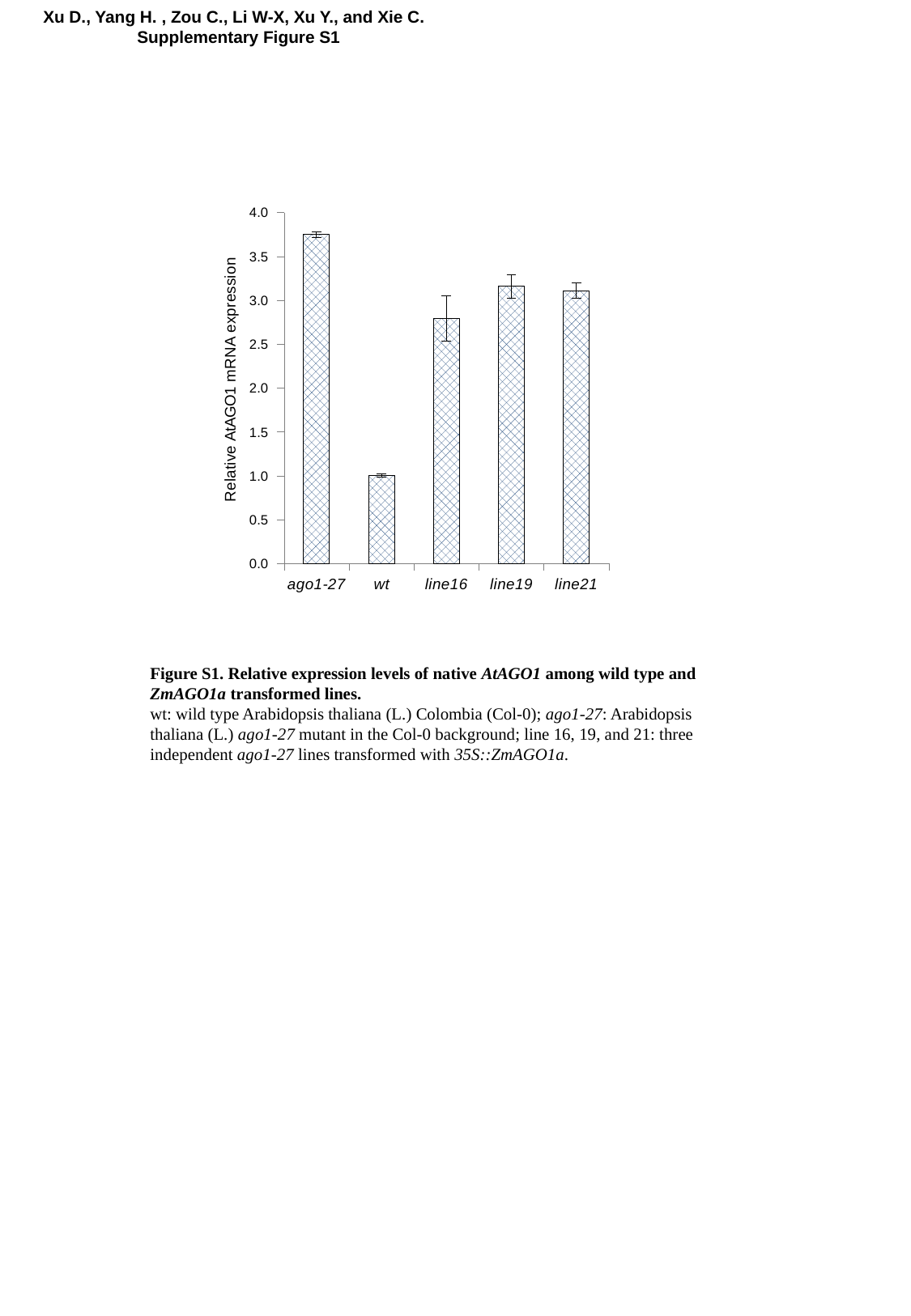

Xu D., Yang H. , Zou C., Li W-X, Xu Y., and Xie C.
Supplementary Figure S1
### Chart
| Category | |
|---|---|
| ago1-27 | 3.74885733343242 |
| wt | 1.0003613668307352 |
| line16 | 2.7947788841595815 |
| line19 | 3.1607908858031 |
| line21 | 3.109486068938731 |Figure S1. Relative expression levels of native AtAGO1 among wild type and ZmAGO1a transformed lines.
wt: wild type Arabidopsis thaliana (L.) Colombia (Col-0); ago1-27: Arabidopsis thaliana (L.) ago1-27 mutant in the Col-0 background; line 16, 19, and 21: three independent ago1-27 lines transformed with 35S::ZmAGO1a.
